# *Aloe vera* Leaf Extract Reduced BBB Permeability and Improved Neurological Results after Traumatic Brain Injury: The Role of Oxidative Stress

**DOI:** 10.1155/2024/5586814

**Published:** 2024-07-15

**Authors:** Mohammad Khaksari, Marzieh Shahryari, Alireza Raji-Amirhasani, Zahra Soltani, Bahram Bibak, Zakieh Keshavarzi, Farzaneh Shakeri

**Affiliations:** ^1^ Endocrinology and Metabolism Research Center Kerman University of Medical Sciences, Kerman, Iran; ^2^ Department of Physiology and Pharmacology Afzalipour Faculty of Medicine Kerman University of Medical Sciences, Kerman, Iran; ^3^ Department of Physiology Neuroscience Research Center Medical Faculty Golestan University of Medical Sciences, Gorgan, Iran; ^4^ Physiology Research Center Institute of Neuropharmacology Kerman University of Medical Sciences, Kerman, Iran; ^5^ Natural Products and Medicinal Plants Research Center North Khorasan University of Medical Sciences, Bojnurd, Iran; ^6^ Department of Physiology and Pharmacology School of Medicine North Khorasan University of Medical Sciences, Bojnurd, Iran

## Abstract

**Introduction:**

Recognizing the importance of medicinal plants and the absence of specific medications for traumatic brain injury (TBI) treatment, this study was conducted to evaluate the effects of an aqueous extract of *Aloe vera* on oxidative stress, blood–brain barrier (BBB) permeability, and neurological scores following TBI.

**Materials and Methods:**

Adult male rats were categorized into five groups: sham, TBI, vehicle, low-dose *Aloe vera* (LA), and high-dose *Aloe vera* (HA). We induced diffuse TBI using the Marmaro model and administered the aqueous *Aloe vera* leaf extract, as well as vehicle, via intraperitoneal injection half an hour after TBI. Neurological outcomes were assessed both before and several hours after TBI. Additionally, oxidative stress factors were measured 24 hr after TBI, and Evans blue content (a BBB permeability index) was determined 5 hr after TBI in both serum and brain.

**Results:**

Both LA and HA reduced the increase in BBB permeability after TBI, with HA having a more pronounced effect than LA. Both *Aloe vera* doses decreased brain MDA levels, increased brain TAC, and lowered both serum and brain PC levels. The impact of *Aloe vera* on brain oxidative parameters was more significant than on serum. HA also counteracted the declining effects of TBI on neurological outcomes at 4 and 24 hr post-TBI.

**Conclusion:**

This study suggests that *Aloe vera* extract may reduce BBB permeability and improve neurological outcomes after TBI by decreasing oxidative factors and increasing antioxidant factors.

## 1. Introduction

Each year, a significant number of individuals worldwide experience traumatic brain injuries (TBI). These injuries are the leading cause of death and disability among young people [1]. In the United States, the emergency department reports more than 2.5 million TBI patients annually [1]. Biological responses following brain injury, resulting in cell death, involve factors such as pathological neuronal stimulation, inflammation, free radical production, and cerebral hemorrhage [2, 3]. Oxidative stress appears to play a key role in neuronal destruction after TBI. Additionally, oxidative compounds harm myelin lipoproteins within axonal structures, besides their detrimental effects on neuronal membranes [4]. Oxygen free radicals' accumulation in arteries significantly contributes to the molecular cascade involved in the disruption of the blood–brain barrier (BBB) [5]. BBB serves as a dynamic functional interface, facilitating the transport of essential nutrients, amino acids, ions, and more between the bloodstream and the central nervous system (CNS). Simultaneously, it acts as a protective shield, preventing the entry of various pathogens and harmful substances [6, 7, 8, 9, 10]. The primary roles of the BBB include the maintenance of brain homeostasis and the safeguarding of the CNS against potential threats, both endogenous and xenobiotic, thereby promoting optimal neuronal function [10, 11, 12]. Typically, the BBB consists of brain endothelial cells, tightly interconnected by junction proteins like cadherins, as well as tight junctional proteins such as occludin and claudins [10, 13]. TBI results in harm to endothelial cells, disrupting blood flow and causing damage to tight junction proteins and the basal membrane. This damage leads to a breakdown in the integrity of the BBB and increased permeability [14], potentially allowing harmful molecules to infiltrate the brain tissue. This breach can trigger inflammatory responses and oxidative stress, exacerbating the damage caused by TBI [10]. Free radicals can also harm endothelial cells, leading to cytotoxic and vasogenic edema [4].

For decades, herbal medicine has been considered a primary source of pain relief. Nowadays, despite the significant advancements in synthetic drugs, medicinal plants and their formulations remain widely used. They are also an integral part of the healthcare system in some countries [15]. One such medicinal plant is *Aloe vera*, which has garnered special attention for centuries due to its numerous medicinal and therapeutic properties [16]. *Aloe vera* belongs to the *Liliaceae* genus and is found in Asia, Africa, and tropical regions across the world [17]. *A. vera* leaves consist of three main parts: the skin, latex, and the gel layers. Studies have revealed that the gel contains more than 200 active ingredients, including essential body elements, vitamins, proteins, fats, amino acids, and polysaccharides [18].

In chronic inflammation, *A. vera* prevents both acute (the first phase) and chronic inflammation (the second phase). The first phase involves the inhibition of mediators like histamine and serotonin, while the second phase involves the inhibition of cyclooxygenase and TGF-*β* release [19]. Many anti-inflammatory drugs interact with oxidants, and it has been reported that oxidant scavengers play a role in the anti-inflammatory action of these drugs [20]. Among *A. vera*'s properties, its antioxidant attributes stand out, which are associated with vitamins A, C, and E, all of which are potent antioxidants. These substances and compounds work by reducing lipid peroxidation and inhibiting the production of free radicals [21, 22].

Several studies have demonstrated the antioxidant and anti-inflammatory properties of *A. vera*. For instance, Gupta et al. [23, 24, 25, 26, 27] recently reported that pesticides, such as Cartap and Malathion, induce oxidative stress and physiological disorders in rats, affecting both the brain and liver. However, *A. vera* extract exhibited significant protective properties against these pesticide-induced toxicities, likely attributed to its antioxidant and anti-inflammatory compounds [23, 24, 25, 26, 27].

The purpose of this study was to investigate the potential effects of an aqueous extract of *A. vera* on various aspects related to traumatic brain injury. Given the lack of specific medications for TBI treatment and the growing importance of medicinal plants, the research aimed to assess the impact of *A. vera* extract on oxidative stress, BBB permeability, and neurological scores following TBI. Ultimately, the purpose of the research was to provide insights into the potential therapeutic effects of *A. vera* extract in the context of TBI management.

## 2. Materials and Methods

### 2.1. Animals and Animal Groups

In this research, we utilized 70 male Wistar rats weighing between 200 and 250 g. The animals were purchased from Kerman University of Medical Science and ranged in age from 11 to 12 weeks. The rats were housed in the Kerman University of Medical Sciences animal facility, maintained at a temperature range of 20–22°C, and subjected to a 12-hr light–dark cycle. They had ad libitum access to both water and food. The study was conducted in compliance with the ethics committee of Kerman University of Medical Sciences under ethics code 95/27.

A total of 70 male Wistar rats were randomly assigned to five groups:

1. Sham group: Rats in this group underwent the surgical procedure for traumatic brain injury (TBI) without actual induction of TBI [28, 29].
2. TBI group: In this group, the animals were administered anesthesia with ketamine and xylazine before undergoing traumatic brain injury [28, 29].
3. Vehicle group (VEH): Rats were intraperitoneally (i.p.) injected with the vehicle, which was distilled water (1 mL/kg), half an hour after the brain trauma [21].
4. Low-dose (LA) group: Animals received an intraperitoneal injection of 200 mg/mL/kg of aqueous *A. vera* leaf extract, administered half an hour after the brain injury [21].
5. High-dose (HA) group: Rats received an intraperitoneal injection of 400 mg/mL/kg of *A. vera* extract, administered 30 min after TBI [21].

Also, before intraperitoneal injection, we performed aspiration to make sure that the needle of the syringe was not in the animal's bladder or blood vessels.

Each group was further subdivided into two subgroups, each consisting of seven rats:

In the first subgroup, we assessed neurological outcomes at various time points before and after TBI using the veterinary coma scale (VCS) assessment. We also measured oxidative and antioxidant factors 24 hr after TBI. In the second subgroup, extravascular Evans blue content (used as a blood–brain barrier permeability index) was measured 5 hr after TBI.

### 2.2. *A. vera* Extract Preparation

Fresh *A. vera* leaves were procured from a local grocery store in Kerman province, originating from the arid regions of Fars province. These leaves underwent scrutiny and validation by a pharmacognosist affiliated with the pharmacy faculty at Kerman University. The process involved removing the outer bulge of the leaves with a knife, followed by cutting the leaves into pieces and thoroughly washing them with cold water. Subsequently, 200 g of the prepared material was blended with 100 mL of distilled water using an electric mixer (manufactured in Iran) for approximately 3 min, resulting in a 200% extract (w/v). The resultant mixture was then filtered through a cloth (2.0 *μ*m) and subjected to vacuum filtration using a lyophilizer (0.22 *μ*m for sterile filtration) (manufactured in Germany). Afterward, it was dried at a temperature of 50°C under sterilized conditions and safely stored in a freezer at −20°C until it was ready for use [19]. Some of the finished products were inoculated onto agar plates to detect the presence of any microbes. The chosen doses for this study were 200 mg/mL/kg (referred to as the low dose) and 400 mg/mL/kg (referred to as the high dose). These doses were determined based on previous research assessing both the toxicity and the effective dosage of *A. vera* [30, 31, 32, 33].

### 2.3. Induction of TBI

The Marmarou impact acceleration model is one of the most widely used methods for inducing diffuse traumatic brain injury (TBI) experimentally. This model attempts to mimic aspects of fall or vehicle accident injuries seen clinically in humans. Typically adult rats weighing 200–250 g are used, as the model causes axonal damage throughout the cerebral cortex and brainstem [34].

In the model, rats are first deeply anesthetized using a ketamine (80 mg/kg)/xylazine (15 mg/kg) cocktail [28]. Then a midline scalp incision is made to expose the skull using aseptic surgical techniques including sterile instruments and gloves. A stainless steel disk measuring 3 mm thick and 10 mm in diameter is firmly affixed to the central area of skull between bregma and lambda using dental cement. All animals underwent intubation before the initiation of TBI. A 16-G angiocatheter was employed for intubation, guided by transillumination. The rat is then positioned on foam padding, and a 300 g weight is allowed to freely fall 2 m through a vertical plexiglass tube directly onto the cemented steel disk. This impacts the head and induces diffuse axonal injury [28, 34].

The trauma triggers widepsread neuronal damage, neuroinflammation, blood–brain barrier disruption, and impairment in neurological function. After injury, the rats are connected to a mechanical ventilator (TSA animal respiratory compact, manufactured in Germany) with settings of tidal volume 6 mL/kg, respiratory rate 70 breaths/min, and FiO_2_ 30%. Once spontaneous breathing returns, the endotracheal tube is removed [35, 36, 37]. Finally, the scalp is sutured closed, and rats are singly housed in cages during recovery [35, 36, 37].

In the current study's TBI procedure, great care was taken to closely follow the methodology previously described by other researchers in implementing the Marmarou model [38]. The process, from initial confirmation of anesthesia by lack of reflex response to tail pinch to actual induction of injury, took approximately 10 min per animal. In all phases of the work and all testing, the experimenters were blinded to the treatment groups.

### 2.4. Veterinary Coma Scale (VCS) Assessment

The VCS is a 15-point quantitative neurological assessment tool used to evaluate the level of consciousness and degree of brain injury in animals. It consists of three subscales evaluating motor (1–8 points), visual (1–4 points), and respiratory (1–3 points) function:

The motor response subscale (1–8 points) is as follows:

  1 point: Complete lack of movement; no response to stimuli
  2 points: Extensor, abnormal posturing either spontaneously or induced by stimuli
  3 points: Spontaneous repetitive pedaling motions
  4 points: Weak withdrawal or pedaling to pinch stimuli
  5 points: Visually tracks with head lift but lacks ability to stand or sit upright
  6 points: Difficulty standing but retains some sternal control
  7 points: Drowsy but has voluntary goal-directed movements
  8 points: Normal motor function and dexterity demonstrated

The visual subscale (1–4 points) is as follows:

  1 point: No eyelid response to stimuli
  2 points: Normal eyelid reflexes
  3 points: Open on stimulation
  4 points: Open

The respiratory subscale (1–3 points) is as follows:

  1 point: Absent pattern/Apnea
  2 points: Severe dysrhythmia
  3 points: Normal respiratory pattern

To conduct VCS scoring, two investigators independently rate motor, visual, and respiratory activity in the rats at designated time intervals. Scoring is based on direct observation of responses in each domain, including provocation through stimuli to elicit movements, visual reactions, or alterations in respiratory patterns. Consensus scores between the two examiners are used for analysis [39, 40]. The overall VCS score ranges from 3 to 15, with a higher score indicating a better neurological prognosis [28]. In our investigation, neurological assessments were conducted at multiple time points, including 2 hr before the trauma, immediately following the trauma, and at 1, 4, and 24 hr posttrauma [28, 35].

### 2.5. Determination of BBB Permeability

To assess the BBB permeability, we quantified extravascular Evans blue (EB) content employing a spectrophotometer. Five hours posttrauma, the BBB permeability was evaluated by administering EB through the vein of tail. Under deep anesthesia with ketamine (80 mg/kg) and xylazine (15 mg/kg) (the depth of anesthesia was confirmed by the absence of a response to a painful stimulus), 20 mg/kg of 2% EB solution (1 mL/kg) was injected via the tail vein at 4 hr after trauma. At 1 hr following injection (at the 5-hr posttrauma mark), we opened the thorax and clamped the descending aorta. Subsequently, we infused 200–300 mL of isotonic saline solution into the left ventricle over 20 min to remove intravascular EB content. To achieve this, we bilaterally cut the jugular vein and continued the infusion until all EB was removed [41]. Following this, we expeditiously removed, weighed, and homogenized the brain. It was then immersed in a 20 mL solution composed of 6 mL of 1% sodium sulfate and 14 mL of acetone and agitated for 24 hr. In the following stage, 1 mL of the supernatant solution was collected and combined with 1 mL of trichloroacetic acid. After cooling for 2–3 min, it was centrifuged at 2,000 cycles/min for 10 min. Following centrifugation at 1,000 *g* for 30 min, we measured the absorbance of EB in the supernatant at 620 nm utilizing a spectrophotometer (UV/VIS, Spectrometer, UK). The quantity of extravasated EB content was quantified as *µ*g/g of brain tissue [41, 42].

### 2.6. Measurement of Serum Concentrations of MDA, TAC, and PC

At the 24-hr mark post-TBI, we anesthetized the animals with ketamine (80 mg/kg) and xylazine (15 mg/kg). Blood samples were directly collected from the rat's heart and then underwent centrifugation using a G-6B Centrifuge (France) to separate the serum. Subsequently, we measured malondialdehyde (MDA), a lipid peroxidation indicator, total antioxidant capacity (TAC), and protein carbonyl (PC), serving as a marker of protein peroxidation, according to the protocols outlined in ELISA kits (Eastibiopharm, USA) [43, 44, 45, 46].

### 2.7. Measurement of Brain Concentration MDA, TAC, and PC

At the 24-hr mark post-TBI, we anesthetized the animals and removed their brains from the skull, immediately freezing them with liquid nitrogen. Brain homogenization was performed using a homogenizer (Heelscher, Germany). For this process, 500 mg of each brain sample was blended with 2 mL of buffer (pH = 7.2) which included 50 mmol Tris, 0.5% Triton 100-X, a protease inhibitor cocktail (Roche, Germany), and 150 mmol NaCl [29].

Subsequently, the homogenized solution underwent centrifugation in a refrigerated centrifuge (Rotina, Germany) at 4,000 rpm and 2°C for 15 min. The resulting supernatant was utilized to measure the brain concentration of antioxidants and oxidants. We analyzed the samples using an ELISA kit following its respective protocol [44, 45, 47, 48].

### 2.8. Statistical Analysis

The results were presented as mean ± SEM. We assessed the data's normal distribution using the Shapiro–Wilk test. When the data followed a normal distribution, we applied the ANOVA test with post hoc Tukey test to compare the means among different groups. For data that did not follow a normal distribution, we employed the Kruskal–Wallis test. Furthermore, we used a *t*-test for comparisons between two parameters. We considered a significance level of *P*  < 0.05 as the minimum threshold for statistical significance. Data analysis was performed using GraphPad Prism 8 [49, 50].

## 3. Results

### 3.1. BBB Permeability (EB Content of the Brain)

**Figure 1:**
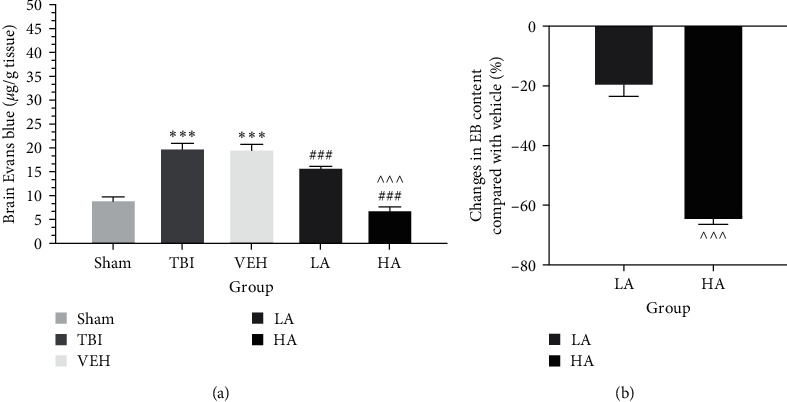
Comparison of Evans blue content (*µ*g/g tissue) in different groups (*n* = 7). Data represented as mean ± SEM. (a)  ^*∗∗∗*^*P* < 0.001 vs. sham, ^###^*P* < 0.001 vs. VEH, and ^^^^^*P* < 0.001 vs. LA. (b) ^^^^^*P* < 0.001 vs. LA. VEH, vehicle; LA, low dose of *A. vera*; HA, high dose of *A. vera*; EB, Evans blue.

The permeability of the BBB is depicted in Figure 1. In the TBI group (20.16 ± 0.9 *μ*g/g) and VEH group (19.81 ± 0.98 *μ*g/g), the EB content showed a significant increase (*P*  < 0.001) compared to the sham group (8.48 ± 0.67 *μ*g/g) (Figure 1(a)). This index was reduced in the LA group (15.96 ± 0.18 *µ*g/g) and HA group (7.06 ± 0.59 *µ*g/g) compared to the VEH group (*P*  < 0.001) (Figure 1(a)). Furthermore, the EB content in the HA group was significantly lower than in the LA group (*P*  < 0.001). The reduction percentage was higher in the HA group (−65.1% ± 1.42%) than in the LA group (−20% ± 3.5%) (*P*  < 0.001) (Figure 1(b)). This indicates that the high dose of *A. vera* induced a greater decrease in BBB permeability compared to the low dose of *A. vera*.

### 3.2. VCS Score in Different Times before and after TBI

**Figure 2:**
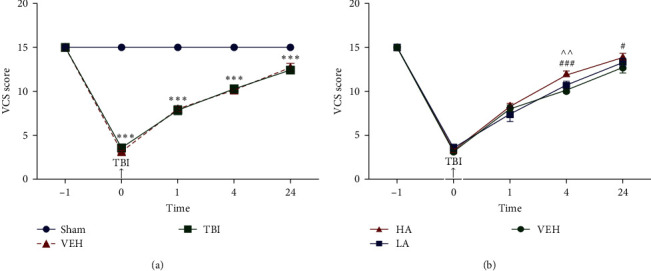
Comparison of veterinary coma scale (VCS) scores at different time points in different groups after TBI (*n* = 7). Data represented as mean ± SEM. (a)  ^*∗∗∗*^*P* < 0.001 vs. sham. (b) ^###^*P* < 0.001 HA vs. VEH at 4 hr, ^^^^*P* < 0.01 HA vs. LA at 4 hr, and ^#^*P* < 0.05 HA vs. VEH at 24 hr. VEH, vehicle; LA, low dose of *A. vera*; HA, high dose of *A. vera*; TBI, traumatic brain injury.

The VCS scores of the experimental groups at various time points before and after TBI are displayed in Figure 2. The VCS scores of different groups were not significantly different 1 hr before TBI. However, immediately after the trauma, the neurological scores in the TBI (3.57 ± 0.29) and VEH (3.14 ± 0.14) groups substantially decreased compared to the sham group (15 ± 0) (*P*  < 0.001). Additionally, VCS scores were lower in the TBI and VEH groups compared to the sham group (*P*  < 0.001) at 1, 4, and 24 hr after brain injury (Figure 2(a)).

VCS scores were not significantly different between the LA, HA, and VEH groups 1 hr before and 1 hr after the trauma. However, a substantial increase in VCS was observed in the HA group (11.85 ± 0.26) compared to the VEH (*P*  < 0.001) and LA (*P*  < 0.01) groups at 4 hr following TBI. At 24 hr after trauma, the VCS score in the HA group (13.85 ± 0.34) showed a significant increase (*P*  < 0.05) compared to the VEH group. However, the VCS score of the LA group was not significantly different from the VEH and HA groups at 24 hr after TBI (Figure 2(b)).

### 3.3. The Serum and Brain TAC Levels after TBI

**Figure 3:**
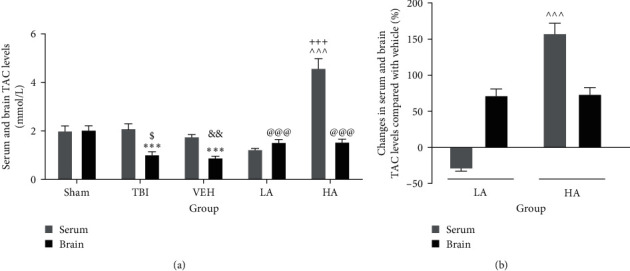
Comparison of TAC changes in serum and brain of different groups (*n* = 7). Data represented as mean ± SEM. (a)  ^*∗∗∗*^*P*  < 0.001 vs. sham brain, ^$^*P*  < 0.05 vs. TBI serum, ^&&^*P*  < 0.01 vs. VEH serum, ^^^^^*P*  < 0.001 vs. VEH and LA serum, ^@@@^ P < 0.001 vs. VEH brain, and ^+++^*P*  < 0.001 vs. HA brain. (b) ^^^^^*P*  < 0.001 vs. LA serum. VEH, vehicle; LA, low dose of *A. vera*; HA, high dose of *A. vera*; TBI, traumatic brain injury; TAC, total antioxidant capacity.

Serum TAC levels were not significantly different in the sham, TBI, and VEH groups. However, the level of TAC in the brains of the TBI (1.03 ± 0.11 mM) and VEH (0.9 ± 0.05 mM) groups was significantly reduced compared to the brain level of the sham group (2.05 ± 0.016 mM) (*P*  < 0.001). The brain level of TAC in the TBI and VEH groups was lower than the serum level of this index (*P*  < 0.05 and *P*  < 0.01, respectively) (Figure 3(a)). Although the brain level of TAC was increased by both doses of *A. vera* (LA and HA) compared with the VEH (*P*  < 0.001), the serum level of this index was higher only in the HA group than in the VEH and LA groups (*P*  < 0.001). Also, the serum TAC level of the HA group was higher than its brain level (*P*  < 0.001) (Figure 3(a)). Additionally, the percentage increase in serum TAC in the HA group (157.92% ± 14.2%) was higher than the LA group (*P*  < 0.001), but no difference in the percentage increase in brain TAC was observed between the two doses (Figure 3(b)).

### 3.4. The Serum and Brain PC Levels following TBI

**Figure 4:**
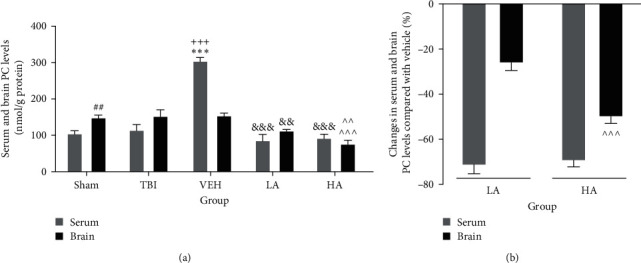
Comparison of PC changes in serum and brain of different groups (*n* = 7). Data represented as mean ± SEM. (a)  ^*∗∗∗*^*P* < 0.001 vs. sham and TBI serum, ^##^*P* < 0.01 vs. sham serum, ^+++^*P*  < 0.001 vs. VEH brain, ^&&&^*P*  < 0.001 vs. VEH serum, ^&&^*P*  < 0.01 vs. VEH brain, ^^^^^*P*  < 0.001 vs. VEH brain, and ^^^^*P*  < 0.01 vs. LA brain. (b) ^^^^^*P*  < 0.001 vs. LA brain. VEH, vehicle; LA, low dose of *A. vera*; HA, high dose of *A. vera*; TBI, traumatic brain injury; PC, protein carbonyl.

Serum PC levels of the VEH group (305.23 ± 8.86 nmol/g protein) were higher than in the sham (105.5 ± 7.26 nmol/g protein) and TBI (115.29 ± 14.21 nmol/g protein) groups (*P*  < 0.001). However, the brain level of this index was not significantly different in the sham, TBI, and VEH groups. The brain PC level was higher in the sham group than the serum PC level in the same group (*P*  < 0.01). Meanwhile, this oxidant was higher in the serum of the VEH group compared to the brain level in the same group (*P*  < 0.001) (Figure 4(a)). The PC serum level in LA (86.85 ± 15.72 nmol/g protein) and HA (93.05 ± 10.20 nmol/g protein) was lower than the serum level of the VEH group (*P*  < 0.001). Additionally, the PC brain level in LA (113.65 ± 2.59 nmol/g protein) and HA (77.06 ± 9.57 nmol/g protein) groups was lower than in the VEH group (*P*  < 0.01 and *P*  < 0.001, respectively). Also, it was lower in the brain of the HA group compared to the LA group (*P*  < 0.01) (Figure 4(a)). The percentage changes in PC serum levels were not significantly different between LA and HA groups. However, the percentage decrease in brain levels of this index in the HA group was greater than in the LA group (*P*  < 0.001) (Figure 4(b)).

### 3.5. The Serum and Brain MDA Levels following TBI

**Figure 5:**
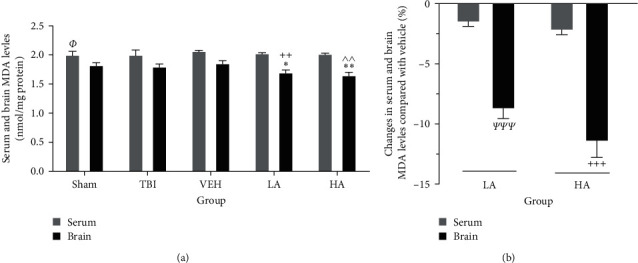
Comparison of MDA level changes in serum and brain of different groups (*n* = 7). Data represented as mean ± SEM. (a) ^*Φ*^*P*  < 0.05 vs. sham brain,  ^*∗*^*P*  < 0.05 vs. VEH brain,  ^*∗∗*^*P*  < 0.01 vs. VEH brain, ^++^*P*  < 0.01 vs. LA serum, and ^^^^*P*  < 0.01 vs. HA serum. (b) ^*ᴪᴪᴪ*^*P*  < 0.001 vs. LA serum and ^+++^*P*  < 0.001 vs. HA serum. VEH, vehicle; LA, low dose of *A. vera*; HA, high dose of *A. vera*; TBI, traumatic brain injury; MDA, malondialdehyde.

The serum MDA levels were not different among the sham, TBI, and VEH groups. Likewise, the brain MDA levels in the sham, TBI, and VEH groups did not show significant differences. However, the serum MDA level in the sham group was higher than that in the brain of the same group (*P*  < 0.05) (Figure 5(a)). Additionally, serum MDA levels in the LA, HA, and VEH groups did not exhibit significant differences. Conversely, there was a decrease in the brain levels of this index in the LA (1.70 ± 0.04 nmol/mg protein) and HA (1.65 ± 0.05 nmol/mg protein) groups compared to the VEH group (*P*  < 0.05 and *P*  < 0.01, respectively). Notably, this level in the brains of the LA and HA groups was lower than their serum MDA levels (*P*  < 0.01) (Figure 5(a)). The percentage of decrease in serum and brain MDA levels in the LA and HA groups did not differ significantly from each other. However, the percentage of decrease in brain MDA level of this index in the LA and HA groups was greater than the percentage decrease in their serum levels (*P*  < 0.001) (Figure 5(b)).

## 4. Discussion

The use of many herbal medicines, in addition to being useful in the treatment of many diseases, also has fewer side effects [51]. Therefore, in the present study, the neuroprotective effect of a medicinal plant called *A. vera* leaves in reducing BBB permeability after TBI was investigated. The most important findings of the present study are as follows:

Both doses of *A. vera* reduce BBB disruption, although HA showing greater effects than LA. After TBI, VCS scores decreased, and this decrease continued for up to 24 hr. However, HA was able to increase VCS scores at 4 and 24 hr. Both doses of *A. vera* were able to reduce brain MDA levels and both serum and brain PC levels. Additionally, these doses prevented a decrease in TAC brain levels. HA also increased TAC serum levels following TBI.

The integrity of BBB function plays a vital role in maintaining normal brain volume and brain homeostasis. Any structural or functional defect in the BBB can result in vasogenic edema [52]. The results of the present study indicate an increase in BBB permeability after TBI, attributed to the disruption of the BBB. In the trauma group, the extravascular EB content increased by 2.5 times. These findings align with previous reports, which have described the rapid onset of injury during the initial phase of TBI, leading to a peak increase in permeability within the first hours after trauma [53]. The maximum permeability typically occurs between 4 and 6 hr after TBI [54]. Previous studies conducted in our laboratory have also confirmed increased BBB permeability in both male [55, 56] and female [42, 57] animals following TBI. Several factors can contribute to the heightened BBB permeability observed after TBI, including BBB and vascular endothelium damage and, in certain instances, increased vesicle movement from the vascular endothelium [58], astrocyte damage [59], and elevated levels of macrophages, microglia, and inflammation [60, 61].

In another aspect of this research, we observed a reduction in EB content by 20% and 65%, respectively, with low and high doses of *A. vera*, which exhibited dose-dependent effects. The aqueous extract of *A. vera* may potentially decrease BBB permeability through the following mechanisms: (1) anti-inflammatory activity attributed to the presence of phenolic compounds in the aqueous extract of *A. vera* [19]; (2) suppression of prostaglandin E2 production, likely due to the presence of vitamins B1, B2, B6, beta-carotene, choline, folic acid, and alpha-tocopherol [19, 62, 63, 64, 65, 66]; and (3) antioxidant properties of *A. vera* [23, 24, 25, 26, 27, 67].

The presence of phenolic compounds in the aqueous extract of *A. vera* [22, 19] contributes to its anti-inflammatory properties, and the suppression of inflammatory processes plays an important role in reducing BBB permeability. Therefore, the existence of these compounds, owing to their anti-inflammatory potential, appears to be a key factor in reducing BBB permeability. Also the presence of essential vitamins (B1, B2, B6, beta-carotene, choline, folic acid, and alpha-tocopherol) in *A. vera* extract introduces another layer of potential influence [22, 26, 62, 63, 64, 65, 66, 19]. These vitamins affect various physiological processes, including inhibiting the production of prostaglandin E2. By doing so, the extract could help to stabilize BBB permeability dynamics, providing another explanation for its observed effects. In addition, experimental TBI data in laboratory animals support the beneficial effects of administering antioxidants in TBI [67, 68], and several studies have also reported that *A. vera* has antioxidant properties [23, 24, 25, 26, 27]. It appears that in our study, the antioxidant properties of *A. vera* were effective in improving the BBB.

The identification and elucidation of the bioactive molecules in the aqueous extract of *Aloe vera*, along with their specific functions, are of great importance. While our study did not address this aspect, future research could unravel the complex network of bioactive components responsible for the observed effects on BBB permeability. This knowledge can open new ways to use the bioactive compounds of *Aloe vera* for better treatments.

Neurological outcomes, as assessed by the VCS score, were also evaluated in this study. It was observed that the VCS score decreased and continued to decline for up to 24 hr after TBI. However, the high dose of the extract increased this index at 4 and 24 hr post-TBI, indicating that the extract's effect was dose-dependent. The mechanisms contributing to the enhanced impact of *A. vera* on neurological outcomes may include the regulation of brain edema and BBB permeability, reduction of inflammatory factors [69], mitigation of oxidative damage in the hippocampus and cortex [21, 70], and improvement in heart function [71]. It is worth noting that, as there were no previous studies on the *A. vera*'s effect on neurological scores following TBI, the exact mechanism remains unknown.

Changes in oxidant and antioxidant factors were examined in another part of this analysis to elucidate the mechanisms underlying the beneficial effects of *A. vera*. TBI led to a reduction in brain TAC levels. However, following *A. vera* consumption, TAC levels increased in both the brain and serum. Notably, both doses of *A. vera* significantly raised brain TAC levels (by 73.64% and 71.81%, respectively), while serum TAC levels experienced an increase only with the high dose. Consequently, it can be inferred that the effect of *A. vera* on brain TAC levels was dose-dependent.

TAC represents a collection of enzymatic and nonenzymatic antioxidants, collectively reflecting the overall antioxidant capacity. These antioxidants function as scavengers of free radicals [72]. Numerous studies have documented a decline in TAC levels in TBI patients [73]. A separate study demonstrated that *A. vera* mitigated oxidative stress in diabetic rats [74]. The potential mechanisms by which *A. vera* augments antioxidant activity, such as TAC, while reducing oxidative factors, may be attributed to the presence of vitamins A, C, and E in the leaves of this plant. All three of these vitamins possess antioxidant properties, acting to reduce lipid peroxidation rates and the generation of free radicals [21, 23, 75]. One limitation of this study was the absence of experiments aimed at identifying the specific compounds within this extract, mainly due to constraints related to financial resources and time.

The study also demonstrated that *A. vera* decreased PC levels following TBI. An elevation in PC levels, considered an indicator of oxidative stress in TBI, was previously reported by Petronilho et al. [76]. Carbonization is essentially an irreversible alteration resulting from oxidative stress, representing the most common and widespread form of protein oxidation [77]. In this study, it was observed that the traumatic brain group exhibited an increase in PC levels.

However, both doses of *A. vera* led to reductions in serum (by 71% and 69%, respectively) and brain (by 26% and 50%, respectively) PC levels. Also, the reason for the high levels of serum PC in the VEH group is unclear. In our studies, no confounding factors were observed, and this group probably had high levels of serum PC compared to other groups due to the solvent used.

While no prior studies have investigated the impact of this extract on TBI and subsequently measured oxidative stress, there is a study indicating that *A. vera* reduced oxidative stress in the brains of diabetic animals by lowering PC levels [70]. This extract, with its antioxidant properties, also safeguards kidney cells and reduces tubular damage induced by oxidative agents [78].

The findings revealed that both doses of *A. vera* reduced brain MDA levels, although these levels were lower in both groups than the serum MDA levels. Oxidative stress plays a crucial role in neuronal cell death following TBI. MDA is the end product of lipid peroxidation induced by ROS, which increases BBB permeability in TBI [74, 79]. One of the potential neuroprotective mechanisms of this extract is perhaps a decrease in MDA and PC levels or an increase in TAC levels.

Brain membrane lipid injury is an early occurrence in TBI, and an increase in MDA levels can be observed as early as 30 min after damage, persisting for up to 72 hr after the injury [80]. Both doses of the extract reduced this oxidant in the brain and serum. In another section of the study's results, it was observed that the impact of varying *A. vera* doses on lowering brain MDA levels exceeded their effect on serum MDA levels.

Various doses of *A. vera* have been reported to exhibit antioxidant activity [25, 26, 27]. Wang et al. [81] reported that this extract reduced the production of lipid peroxidation (MDA) in the mitochondria of brain cells. *A. vera* leaf extract has been reported to reduce the progression of kindling [82] and Cartap-induced toxicity by decreasing MDA levels and increasing GSH levels in the brain [25]. Tabatabai et al. reported that *A. vera* protected hippocampal neurons and corrected behavioral problems in diabetic animals by decreasing MDA and increasing SOD [83]. Additionally, Subbia showed that *A. vera* extract reduced glutathione, superoxide dismutase (SOD), catalase, glutathione peroxidase (GP), and glutathione S-transferase in the kidneys and livers of diabetic rats [84].

In summary, numerous studies have reported that during TBI, oxidative stress, and changes in its markers (MDA, TAC, PC, and SOD) are associated with the severity of BBB damage and the deterioration of neurological outcomes [85, 86, 87]. For example, oxidative stress has been reported to activate matrix metalloproteinases (MMPs) and aquaporin 4, as well as downregulate tight junction proteins, ultimately increasing vascular permeability and BBB disruption, leading to increased edema [88]. These reports are consistent with our study, suggesting that the antioxidant properties of the extract are associated with reduced BBB damage and improved neurological outcomes.

The use of male rats was one of the limitations of this study, because due to the limited budget, we could not consider both sexes. However, female sex hormones may protect the brain after injury [89, 90, 91]. Future studies should investigate whether *Aloe vera* works equally well in both males and females. We hope that further work can clarify sex-based differences in herbal treatments for brain injuries. Also, it seems that the solvent itself could have caused the PC increase, and the decreasing effects seen by the LA and HA doses have inhibited both the TBI-induced increase and solvent-induced increase, which indicates the high efficacy of these drugs in preventing a PC increase. Although in subsequent studies that will measure PC, we would not use this solvent and will use a substitute solvent to prevent these confounding factors.

## 5. Conclusion

The aqueous extract of *A. vera* has demonstrated a neuroprotective effect following TBI. Both high and low doses of this extract were found to decrease BBB permeability and increase neurological scores in male rats after TBI. The potential mechanism of action for *A. vera* involves reducing the levels of oxidizing agents, such as PC and MDA, in both the brain and serum, while simultaneously increasing antioxidant agents like TAC.

For future studies, it is recommended to identify the active ingredient(s) within this plant extract. Additionally, exploring other potential mechanisms underlying the extract's neuroprotective effects, such as redox signaling, would be valuable avenues for further research.

## Data Availability

The datasets used during the current study are available from the corresponding author on reasonable request.
